# The Association Between Subjective Hearing Difficulty and Cognitive Impairment: A Stratified Analysis

**DOI:** 10.1093/geroni/igaf122.4022

**Published:** 2025-12-31

**Authors:** Dukyoo Jung, Leeho Yoo, Soo Gyung Shin, Sukyung Byeon, Hyein Seo

**Affiliations:** Ewha Womans University, Seoul-t’ukpyolsi, Republic of Korea; Ewha Womans University, Seoul-t’ukpyolsi, Republic of Korea; Ewha Womans University, Seoul-t’ukpyolsi, Republic of Korea; Ewha Womans University, Seoul-t’ukpyolsi, Republic of Korea; Ewha Womans University, Seoul-t’ukpyolsi, Republic of Korea

## Abstract

Age-related hearing loss (ARHL) affects 50% of older adults, and has become a global concern due to its adverse impact on cognitive function. However, the effects of ARHL within specific subgroups remain unclear. This study aimed to examine the association between ARHL and cognitive impairment (CI) across diverse subgroups of community-dwelling older adults. This is a cross-sectional study using data from the 2023 National Survey of Older Koreans. Hearing-related characteristics included ARHL diagnosis, subjective hearing difficulty, and hearing-aid (HA) use. Complex sample logistic regression was done to examine the relationship between hearing-related characteristics and CI. A total of 7,419 participants were included in the analysis. Among all participants, subjective hearing difficulty (OR = 3.139, p<.001) and HA use (OR = 0.76, p=.044) were both significantly associated with CI in the unadjusted model, while diagnosis of ARHL was not (OR = 1.337, p=.116). These associations remained significant after adjusting for covariates but were attenuated. After stratifying participants by HA use, frailty, sex, age, depression, and social participation, subjective hearing difficulty was significantly associated with CI in the unadjusted model. In the adjusted model, this association remained consistent in all subgroups except for the HA user group. Our findings suggest that subjective hearing difficulty is significantly associated with CI in older adults, even after controlling for covariates. The absence of an association between ARHL diagnosis and CI may reflect limited awareness of ARHL among older adults. Also, stratified analysis suggests the potential preventive role of hearing aids in cognitive decline.

